# A Mixed-Method Analysis of Inequalities Associated With Adverse Sexual and Reproductive Health Outcomes and the Requisite Interventions Among Young Women in Durban Informal Settlements, South Africa

**DOI:** 10.3389/fpubh.2022.810216

**Published:** 2022-02-28

**Authors:** Obasanjo Afolabi Bolarinwa, Tlou Boikhutso

**Affiliations:** Department of Public Health Medicine, School of Nursing and Public Health, University of KwaZulu-Natal, Durban, South Africa

**Keywords:** mixed-method analysis, inequality, adverse sexual and reproductive health outcomes, informal settlements, South Africa

## Abstract

**Background:**

Over the years, positive sexual and reproductive health (SRH) outcomes have been made possible by a series of policies such as the Sustainable Development Goals, targeted toward different aspects of young women's SRH needs. Nevertheless, inequalities in the levels and trends of adverse SRH outcomes still exist in sub-Saharan Africa (SSA), including South Africa. Thus, this study examines the inequalities associated with adverse SRH outcomes among young women in Durban informal settlements, South Africa, using a mixed-method analysis and suggested requisite interventions to reduce or eliminate the disparity.

**Methods:**

A mixed-method sequential explanatory design was used to address the research question. First, a quantitative cross-sectional survey was conducted among 547 young women aged 18 to 24 years in four informal settlements in Durban, South Africa, between April and July 2021. Thereafter, twenty (20) key informant interviews were conducted among different participants but with the same study characteristics. The study's outcome variable was adverse SRH outcomes, including HIV, STIs and unintended pregnancy, while the independent variable was inequality. The quantitative analysis employed binary and multivariable analysis to determine the association between the outcome and explanatory variables, using an alpha level of *p* < 0.05 to determine significance, while the qualitative analysis was done thematically.

**Results:**

At the quantitative level, the prevalence of adverse SRH outcomes among young women dwelling in Durban informal 242 settlements were 17.55%, 9.14% and 18.10% for STIs, HIV and unintended pregnancy, respectively. The adjusted odds ratio showed that young women who ever discussed sexual matters with their parents had a lower likelihood of reporting having STIs [aOR = 0.20; 95% (CI = 0.15–1.01)], HIV [aOR = 0.20; 95% (CI = 0.15–1.01)] and unintended pregnancy [aOR = 0.20; 95% (CI = 0.15–1.01)] compared to young women who never had a sexual discussion with their parents. Almost all the key informant interview participants shared the same perspective and proffered possible solutions in the qualitative results.

**Conclusion:**

There are disparities in the factors associated with adverse SRH outcomes in Durban's informal settlements. Healthcare proximity, child support grants, cigarette smoking, alcohol consumption, polygamous family structures and gender based violence were associated with higher odds of reporting adverse SRH outcomes.

## Background

Sub-Saharan Africa (SSA) has made significant progress in sexual and reproductive health (SRH) outcomes among adolescents and young women over the last few years. Evidence has shown that contraceptive prevalence among single young women (i.e., 15–24 years old) increased by 10%, reflecting an increase from 23% in 1996–2000 to 33% in 2011–2015 (1). In addition, there has been a substantial decline in child marriage incidence across the region (2). Melesse et al. (3) also argued that the HIV epidemic among young people appears to be stalled in most SSA countries, although this is not universal. Positive SRH outcomes over the years have been made possible by a series of policies such as the International Conference on Population and Development's Programme of Action (ICPD-PoA), the Millennium Development Goals (MDGs) and the Sustainable Development Goals (SDGs), targeted toward a different aspect of young women's SRH needs (4).

Nevertheless, there are still inequalities in the levels and trends of adverse SRH outcomes in SSA. For instance, while the highest rate of child marriages is reported in West African countries, the burden of HIV infections abounds in Southern African countries (5, 6), thus raising research interest in exploring the inequalities in SRH outcomes in SSA.

It is important to note that inequalities in SRH outcomes exist at the regional level and within the individual country level. In this study, the focus is centered on informal settlements in South Africa and the inequalities in SRH outcomes in these settlements. Generally, SRH outcomes in South Africa are not homogeneous. The country is overwhelmed with HIV infections and remains the global epicenter of the HIV pandemic (7). Moreover, the 2016 South Africa Demographic and Health Survey (SADHS) revealed that most women aged <22 years had unwanted pregnancies (8). Similarly, Haffejee et al. (9) also reported that the prevalence of unintended pregnancies in South Africa stood at 63%. This shows the heterogeneity in SRH outcomes among young women in South Africa.

Informal settlements in South Africa are continuously growing, with one out-of-seven households in South Africa living in informal settlements (10). Most informal settlements are characterized by high levels of poverty and relative deprivation, with women who find themselves in such settlements being at high risk of adverse SRH outcomes, including HIV infection and STIs, as well as unintended pregnancies (11, 12). However, it is important to note that adverse SRH outcomes are not homogenous within informal settlements. Some young women in informal settlements are more likely than others to have adverse SRH outcomes. For instance, a study in two informal settlements in Nairobi, Kenya, showed that adolescent girls and young women (AGYW) who experienced some form of sexual and gender-based violence or lacked parental support were most likely to report having unintended pregnancies (12). This is consistent with another study conducted in Malawi that also revealed that young women in informal settlements are at high risk of unintended pregnancies (13).

Since 2015, South Africa government and United Nations (UN) member states have signed the 17 SDGs. The SDGs propose to leave no one behind in any of the 17 SDG targets (3). It is imperative to identify and understand the inequalities in term of adverse SRH outcomes (i.e., HIV infection, STIs and unintended pregnancies). However, this subject has not received the attention it requires in South Africa, especially in informal settlements within the country. The question remains: to what extent do inequalities relating to contextual factors, social inequality and parental connectedness influence adverse SRH outcomes? And what are the required social and behavioral interventions to reduce the inequality of adverse SRH outcomes among young women in Durban informal settlements, South Africa? To address these questions, the current study explores adverse SRH outcomes inequality among young women in Durban informal settlements, South Africa, while suggesting requisite social and behavioral interventions to reduce the disparity.

## Methods

### Study Population and Research Design

This study was conducted in four informal settlements in Durban, eThekwini Municipality, KwaZulu-Natal. Durban is the third-most populous city in South Africa with over three million residents, and it is the largest city in KwaZulu-Natal, South Africa. Located on the east coast of South Africa, Durban is the busiest port in the country (14). In the KwaZulu-Natal province, about 239,000 households live in informal settlements. Moreover, over 500 informal settlements are in the eThekwini District, with 20 informal settlements in Durban (15). The study setting was purposively chosen because of available evidence on the high prevalence of adverse SRH outcomes, including STIs, HIV and unintended pregnancy, in KwaZulu-Natal (16).

A mixed-method sequential explanatory design was used to address the research question. First, a quantitative cross-sectional survey was conducted in four informal settlements in Durban in the eThekwini Municipality of the KwaZulu-Natal province, South Africa. After that, key informant interviews were conducted among the same category of respondents but with different participants in order to seek solutions to the SRH outcomes inequalities facing their age group dwelling in informal settlements (17). Living in urban slums or informal settlements has been reported as one of the factors associated with limited access to SRH services (18), with several adverse SRH outcomes being reportedly high among young women residing in informal settlements in South Africa (19–21).

Umgudulu and Banana City informal settlements have 560 and 455 households, respectively, with single-room houses, alongside shacks. These settlements are located 3 km apart from each other, which is ~15 min' drive to the center of Durban. Basic amenities are lacking, and there are no inside toilets or playgrounds for children. The Quarry Road and New Germany informal settlements have 1,200 and 980 households, respectively. They are both located along the main road and are significantly larger than the Umgudulu and Banana City informal settlements in landmass. Pathways are not well-defined or tarred, with the central toilet and laundry washing system being practiced with the shack housing clustered together.

### Quantitative Data Collection and Sample Size Determination

Two research assistants with at least a college certificate were recruited at each informal settlement for this study, these two research assistants were trained explicitly for 1 week on a self-administered questionnaire using the Android version of the Open Data Kit (ODK) mobile application. They were also briefed on the study's objectives. The research assistants were fluent in the widely spoken local language (isiZulu), and all data collection was done under the supervision of the principal investigator. The ODK mobile application simultaneously allows multi-users, ensures privacy, and collects data (22). Android mobile telephones were given to the research assistants by the principal investigator, and these telephones were retrieved after the daily debriefing sessions. The respondents' responses were archived to the server database daily and were deleted from the mobile device before returning the telephone to the research assistants the following day to continue the data collection until the last day of the survey. The data collection was conducted between April–July 2021.

A multi-stage sampling method was employed in the study settings (23). First, all informal settlements in Durban were counted and listed. Thereafter, all the informal settlements' names were written on a different piece of paper, and a simple random sampling technique was used in selecting four informal settlements through ballot (24). At the end of the exercise, the four informal settlements selected were Banana City, Umgudulu, New Germany and Quarry Road.

The researchers employed a systematic random sampling method in selecting the study participants. A list of all shacks/houses was accessed at each study setting through the community leader. Each shack is usually occupied by one household. All households were listed, and the first household was automatically selected as the first eligible household, followed by the next two households, i.e., k = 2 (This is derived by dividing the minimum number of households in a settlement by the desired sample size [i.e. = 455/200]). All eligible young women aged 18–24 years in all the selected households in each informal settlement were interviewed (25).

The minimum sample size should be 420 participants, using Yamane's formula sample size estimation technique at a ±5% precision level and a 95% confidence level (26). However, the study sample size was increased to 800 participants to enable the authors to account for non-response or oversampling errors (27, 28). After considering respondents who are sexually active in line with the study's objective, 547 potential respondents were eligible for this study. As such, the data analysis for this study was limited to the 547 eligible respondents, with 178, 120, 104, 145 respondents from Quarry Road, New Germany, Umgudulu and Banana City, respectively. Prior to data collection, the data collection instrument (a questionnaire scripted on the ODK mobile application) was administered to 20 young women between the ages of 18–24 years old residing in Durban and purposively chosen for the pilot survey/pre-test exercise to validate the instrument.

### Quantitative Analysis and Measures

#### Outcome Variable

The study outcome variable was adverse SRH outcomes measured by HIV, STIs, and unintended pregnancy (29–31). To determine if a participant has ever had HIV/STIs or unintended pregnancy, questions about whether the respondent has ever used medications to treat STIs/tested positive for HIV or whether the respondent has ever had an unintended pregnancy were asked. A binary response of “No” or “Yes” was used to categorize participants' responses. To ensure that the responses supplied by the respondents were correct, a retrospective question was asked as a follow-up question during the interview.

#### Independent Variables

The key independent variables were inequalities derived from contextual factors, including GBV; proximity to a healthcare facility; social inequalities including child support grants and the household or relative wealth index; patterns of behavior, including alcohol intake and smoking of cigarettes; parental connectedness, including parental living arrangements and the discussion of sexual matters with the parent. Other included explanatory variables were family structure, place of settlement, and ethnicity.

GBV was determined by asking if the respondent had ever experienced intimate partner violence, household violence, or any other physical violence exhibited by someone else (32); while healthcare facility proximity was determined by asking the distance of the closest government-owned healthcare facility to the participant's informal settlement (33). GBV was categorized as binary responses “No” or “Yes,” while responses for healthcare facility proximity were category as “ <5 km”, “5 to 9 km,” “10 to 19 km and 20 km and above” (34, 35). The child support grant was determined by asking if the respondent currently receives any grant support toward the upkeep of her child(ren) (36), while the household/relative wealth index was determined by using the household items available in each participant household. The index was constructed in line with demographic health survey (DHS) metrics by assigning a generic number of “1” to each household item such as “electricity, radio, television, mobile telephone, non-mobile telephone, refrigerator, cable TV, generating set, air conditioner, computer, electric iron, fan” listed on the questionnaire. If a respondent did not have any of the listed household items, then it was coded “0.” Based on the household items each respondent had, the researchers then re-categorized the response as “Lower quintile” “Middle quintile” and “Upper quintile” (37). Child support grant responses were categorized as binary “No” or “Yes,” while pattern of behavior was measured by whether the respondent ever drinks alcohol and ever smokes cigarettes, while parental connectedness was measured by whether the respondent ever had a discussion about sexual matters with either of the parents, and whether the respondent was currently living with either of the parents or somewhere else. All the responses for the four variables were categorized in a binary form of “No” or “Yes” (38–41).

Other independent variables included in this study were family type/structure, place of settlement and ethnic group. Family type/structure responses were categorized as either “Monogamous” or “Polygamous.” The place of settlement was the respondent's current informal settlement name, which could be either “Umgudulu,” “Banana City,” “Quarry Road,” or “New Germany.” This was followed by ethnic groups, categorized as “isiZulu,” “Xhosa,” “Sesotho,” and “Siswati.” All selected covariates were based on existing literature (19, 42).

### Quantitative Statistical Analysis

The analysis started with all the 800 young women interviewed. However, after dropping 253 respondents who were not sexually active, the sample size was reduced to 547 sexually active young women. Therefore, this current study's analysis included 547 sexually active young women. First, descriptive statistics were calculated for all the variables included in this study, followed by a chi-square to test the association between the key independent variables, the covariates and outcome variables. Furthermore, a-two logistic regression models were fitted to determine the likelihood of association between selected inequalities such as GBV; proximity to a healthcare facility; social inequalities; patterns of behavior; and parental connectedness. and other independent variables such as family structure, place of settlement, and ethnicity, and the outcome variable (adverse SRH outcomes). The first model (Model I) is a binary logistic regression model, which is an estimate of unadjusted odds ratios of the association between child grant support, wealth index (social inequalities), covariates and HIV/STIs and unintended pregnancy (adverse SRH outcomes). The second model (Model II) is a multivariable model, which included all the covariates, key independent variables and outcome variables considered in this study. The precision level of Alpha <0.05 was considered statistically significant. The multicollinearity test, which used the variance inflation factor (VIF), revealed no evidence of collinearity among the key independent variables and covariates. All the analyses were carried out using Stata version 17.0 (Stata Corporation, College Station, TX, USA).

### Qualitative Interviews

#### Sample Size and Thematic Analysis

Five key informant interviews were conducted in each informal settlement after the quantitative data collection was analyzed and SRH outcomes inequality was determined. Five participants who did not participate in the quantitative research data collection were interviewed in English from each of the four informal settlements, giving a total of 20 participants who suggested interventions that would reduce or eliminate the SRH outcomes inequality discovered in the quantitative data analysis.

The participants were purposively chosen at random. Furthermore, the only criteria observed were that they must not have participated in the earlier quantitative data collection. The interview lasted 20–30 min per participant. A tape recorder and question guide were used during the interviews, conducted by the principal investigator in a private room. All recorded interviews were transcribed thematically using NVivo software. All participants were assigned a unique reference code that has a combination of their informal settlement name and serial number. These unique reference codes were articulated before each interview (43).

Thematic Analysis followed six steps (familiarization, coding, generating, reviewing, naming, defining, and finally, writing up the data analysis). The audiotaped transcripts from the interviews were transcribed verbatim by the principal investigator, and these were read and validated by the academic supervisor. All coding was carried out by highlighting relevant words using sentences or phrases from the transcripts. Each selected inequality was discussed, and suggested solutions were thematically summarized to tackle the variables associated with the inequality of adverse SRH outcomes among young women in Durban informal settlements. The variables include gender-based violence, healthcare proximity, grant child support, pattern of behavior and parental connectedness. The interview exercise, the reporting and transcription follow the approved Consolidated Criteria for Reporting Qualitative Research (COREQ) (44).

## Results

### Quantitative Analysis

#### Descriptive Statistics and Tests of Chi-Square of the Independent and Dependent Variables

The prevalence of adverse SRH outcomes among young women dwelling in Durban informal 242 settlements were 17.55%, 9.14% and 18.10% for STIs, HIV and unintended pregnancy, respectively.

Young women who had ever experienced GBV; those who lived within 10 to 19 km proximity to a healthcare clinic; and those who received child support grants had a 76.92, 83.33, and 70.37% prevalence of unintended pregnancy, HIV, and STIs, respectively. In the same vein, young women dwelling in Durban informal settlements within the Upper quintile; those who drink alcohol; and smoke cigarettes, had a 24.10, 76.22, and 37.68% prevalence of STIs, HIV, and unintended pregnancy.

The test of chi-square showed an association between all selected inequalities and adverse SRH outcomes (STIs, HIV and unintended pregnancy) as *p(x*^2^*)*<* 0.05*, except sexual discussion and unintended pregnancy and ethnicity affiliation and STIs (Table 1).

**Table 1:** Distribution of selected inequalities and adverse sexual and reproductive health outcomes of young women in Durban informal settlements.

**Variables *n* = 547**	**Frequency**	**Percentage**	**Adverse sexual and reproductive health outcomes**					
**Inequalities**			**STIs**		**HIV**		**Unintended pregnancy**	
			**No**	**Yes**	**No**	**Yes**	**No**	**Yes**
**Contextual factors**								
Experienced GBV			*p* (χ^2^) <0.001		*p* (χ^2^) <0.001		*p* (χ^2^) <0.001	
No	404	73.86	90.59	9.41	95.79	90.59	9.41	95.79
Yes	143	26.14	59.44	40.56	76.92	59.44	40.56	76.92
Healthcare proximity			*p* (χ^2^) <0.001		*p* (χ^2^) <0.001		*p* (χ^2^) <0.001	
Less than 5 km	310	56.67	91.94	8.06	98.39	91.94	8.06	98.39
5 to 9 km	208	38.03	68.75	31.25	79.33	68.75	31.25	79.33
10 to 19 km	24	4.39	83.33	16.67	95.83	83.33	16.67	95.83
20 km and above	5	0.91	60.00	40.00	80.00	20.00	80.00	20.00
**Social inequalities**								
Child support grant			*p* (χ^2^) <0.001		*p* (χ^2^) <0.001		*p* (χ^2^) <0.001	
No	439	80.26	95.44	4.56	94.76	95.44	4.56	94.76
Yes	108	19.74	29.63	70.37	75.00	29.63	70.37	75.00
Wealth index			*p* (χ^2^) <0.001		*p* (χ^2^) <0.001		*p* (χ^2^) <0.001	
Lower quintile	310	56.67	88.52	11.48	93.39	88.52	11.48	93.39
Middle quintile	208	38.03	80.43	19.57	89.32	80.43	19.57	89.32
Upper quintile	24	4.39	75.90	24.10	89.16	75.90	24.10	89.16
**Pattern of behavior**								
Drink alcohol			*p* (χ^2^) <0.01		*p* (χ^2^) <0.01		*p* (χ^2^) <0.05	
No	362	66.18	85.64	14.36	93.37	85.64	14.36	93.37
Yes	185	33.82	76.22	23.78	85.95	76.22	23.78	85.95
Smoke cigarettes			*p* (χ^2^) <0.001		*p* (χ^2^) <0.001		*p* (χ^2^) <0.001	
No	478	87.39	85.36	14.64	92.68	85.36	14.64	92.68
Yes	69	12.61	62.32	37.68	78.26	21.74	62.32	37.68
**Parental connectedness**								
Discuss sexual matter			*p* (χ^2^) <0.05		*p* (χ^2^) <0.01		*p* (χ^2^)=0.09	
No	328	59.96	85.06	14.94	88.41	85.06	14.94	88.41
Yes	219	40.04	78.54	21.46	94.52	78.54	21.46	94.52
Living arrangement			*p* (χ^2^) <0.01		*p* (χ^2^) = 0.13		*p* (χ^2^) <0.01	
Not together	339	61.97	79.06	20.94	89.38	79.06	20.94	89.38
Living together	208	38.03	87.98	12.02	93.27	87.98	12.02	93.27
Family structure			*p* (χ^2^) <0.001		*p* (χ^2^) <0.001		*p* (χ^2^) <0.001	
Monogamous	497	90.86	85.31	14.69	93.56	6.44	85.71	14.29
Polygamous	50	9.14	54.00	46.00	64.00	36.00	44.00	56.00
Place of settlement			*p* (χ^2^) <0.001		*p* (χ^2^) <0.001		*p* (χ^2^) <0.001	
Umgudulu	104	19.01	87.50	12.50	95.19	4.81	90.38	9.62
Banana City	145	26.51	59.31	40.69	72.41	27.59	57.24	42.76
Quarry road	178	32.54	87.64	12.36	98.88	1.12	87.64	12.36
New Germany	120	21.94	98.33	1.67	97.50	2.50	95.83	4.17
Ethnicity			*p* (χ^2^) = 0.29		*p* (χ^2^) <0.05		*p* (χ^2^) <0.05	
isiZulu	265	48.45	85.66	14.34	94.34	5.66	86.79	13.21
Xhosa	255	46.62	79.22	20.78	87.06	12.94	76.86	23.14
Sesotho	22	4.02	81.82	18.18	95.45	4.55	81.82	18.18
Siswati	5	0.91	80.00	20.00	80.00	20.00	80.00	20.00
**Overall**			**82.45**	**17.55**	**90.86**	**9.14**	**81.90**	**18.10**

*p (χ^2^) = chi-square p-value*.

#### Binary and Multivariable Logistics Regression Analyses

Table 2 below shows the significant association between selected inequalities and adverse SRH outcomes (STIs, HIV, and unintended pregnancy). All the selected inequalities variables, except the wealth index, showed a significant association with at least one adverse SRH outcome (Model II). The associated inequalities to SRH outcomes included contextual factors (GBV and healthcare proximity), social inequality (child support grant), the pattern of behavior (drink alcohol and smoke cigarettes), parental connectedness (discuss sexual matters and living together with parent) and others (including family structure, place of settlement, and ethnicity).

**Table 2:** Binary and Multivariable logistic regression analysis of selected inequalities and adverse Sexual and Reproductive Health outcomes.

**Variables *n* = 547**	**Adverse sexual and reproductive health outcomes**					
**Inequalities**	**STIs**		**HIV**		**Unintended pregnancy**	
	**Model I** **cOR [95% CI]**	**Model II** **aOR [95% CI]**	**Model I** **cOR [95% CI]**	**Model II** **aOR [95% CI]**	**Model I** **cOR [95% CI]**	**Model II** **aOR [95% CI]**
**Contextual factors**						
**Experienced GBV**						
No	1	1	1	1	1	1
Yes	6.57^***^ [4.10–10.54]	2.26 [0.85–6.02]	6.83^***^ [3.67–12.72]	1.24 [0.50–3.06]	6.39^***^ [4.01–10.19]	2.58^*^ [1.05–6.33]
**Healthcare proximity**						
Less than 5 km	1	1	1	1	1	1
5 to 9 km	5.18^***^ [3.13–8.57]	1.74 [0.39–7.78]	15.90^***^ [6.18–40.91]	7.10^*^ [1.29–39.06]	4.89^***^ [3.01–7.94]	1.66 [0.41–6.76]
10 to 19 km	2.28 [0.72–7.19]	1.06 [0.14–7.85]	2.65 [0.30–23.66]	1.41 [0.96–20.79]	0.91 [0.20–4.10]	0.29 [0.03–3.08]
20 km and above	7.6^*^ [1.21–47.63]	43.19^**^ [3.24–56.38]	15.25^*^ [1.43–161.96]	26.92^*^ [1.41–513.05]	2.52 [0.27–23.31]	6.85 [0.41–114.61]
**Social inequalities**						
**Child support grant**						
No	1	1	1	1	1	1
Yes	49.76^***^ [27.04–91.55]	54.45^***^ [25.01–118.51]	6.03^***^ [3.29–11.04]	2.94^**^ [1.34–6.42]	26.04^***^ [15.09–44.95]	24.39^***^ [12.22–48.67]
**Wealth index**						
Lower quintile	1	1	1	1	1	1
Middle quintile	1.88^*^ [1.09–3.23]	1.20 [0.49–2.94]	1.87 [0.91–3.83]	1.06 [0.42–2.69]	1.86^*^ [1.09–3.17]	1.19 [0.52–2.69]
Upper quintile	2.45^*^ [1.24–4.82]	2.23 [0.76–6.50]	1.90 [0.76–4.78]	1.21 [0.39–3.75]	2.32^*^ [1.19–4.55]	2.28 [0.85–6.09]
**Pattern of behavior**						
**Drink alcohol**						
No	1	1	1	1	1	1
	1.86^**^ [1.19–2.91]	1.01 [0.39–2.59]	2.30^**^ [1.28–4.14]	1.07^*^ [0.49–2.34]	1.74^*^ [1.12–2.71]	0.75 [0.32–1.73]
**Smoke cigarettes**						
No	1	1	1	1	1	1
Yes	3.52^***^ [2.03–6.10]	2.07^*^ [0.71–5.99]	3.51^***^ [1.80–6.85]	1.47 [0.60–3.57]	3.35^***^ [1.94–5.80]	1.72 [0.65–4.56]
**Parental connectedness**						
**Discuss sexual matter**						
No	1	1	1	1	1	1
Yes	0.42^***^ [0.10–1.50]	0.18^*^ [0.10–2.74]	0.44^*^ [0.22–0.87]	0.40^*^ [0.17–0.97]	0.45^**^ [0.39–1.25]	0.03^*^ [0.01–2.11]
**Living arrangement**						
Not together	1	1	1	1	1	1
Living together	0.51^**^ [0.31–0.84]	0.22^**^ [0.09–0.57]	0.61 [0.32–1.15]	0.63 [0.26–1.52]	0.52^**^ [0.32–0.85]	0.26^**^ [0.11–0.59]
**Family structure**						
Monogamous	1	1	1	1	1	1
Polygamous	4.95^***^ [2.69–9.10]	1.53 [0.51–4.59]	8.17^***^ [4.14–16.13]	2.41^*^ [1.02–5.69]	7.63^***^ [4.14–14.09]	3.55^*^ [1.34–9.42]
**Place of settlement**						
Umgudulu	1	1	1	1	1	1
Banana City	4.80^***^ [2.46–9.37]	5.99^**^ [1.94–18.44]	7.54^***^ [2.86–19.88]	3.32^*^ [1.12–9.81]	7.02^***^ [3.38–14.57]	5.91^**^ [2.05–17.04]
Quarry road	0.99 [0.47–2.05]	4.02 [0.60–27.12]	0.22 [0.04–1.18]	0.91 [0.09–8.96]	1.33 [0.60–2.92]	3.47 [0.58–20.78]
New Germany	0.12^**^ [0.03–0.54]	0.79 [0.07–8.45]	0.51 [0.12–2.18]	2.83 [0.33–23.92]	0.41 [0.13–1.24]	3.37 [0.51–22.32]
**Ethnicity**						
isiZulu	1	1	1	1	1	1
Xhosa	1.23 [0.15–10.16]	1.26 [0.58–2.75]	0.42 [005–3.58]	2.92^**^ [1.34–6.32]	1.25 [0.15–10.33]	1.97^*^ [0.98–3.98]
Sesotho	3.58 [0.41–30.97]	3.52 [0.73–16.91]	2.1 [0.24–18.45]	1.35 [0.14–12.67]	3.83 [0.44–33.11]	2.63 [0.57–12.01]
Siswati	2.50 [0.24–25.72]	0.58 [0.00–144.94]	5.09 [0.52–50.00]	2.04 [0.07–56.66]	3.23 [0.32–32.48]	0.70 [0.00–195.56]

* *p < 0.05;*

** *p < 0.01;*

*** *p < 0.001; 1, reference category; cOR, crude odds ratio; aOR, adjusted odds ratio; Model I, unadjusted logistic model; Model II, adjusted logistic model of all the selected inequalities; STIs, sexually transmitted infections; HIV, human immunodeficiency virus*.

The results from the adjusted model (Model II) show that young women dwelling in Durban informal settlements with healthcare proximity between 5 and 9 km [aOR = 43.19; 95% (CI = 3.24–456.38)] who receive child support grants [aOR = 54.45; 95% (CI = 25.01–118.51)], smoke cigarettes [aOR = 2.07; 95% (CI = 0.71–5.99)] and reside in Banana City [aOR = 5.99; 95% (CI = 1.94–18.44)] were more likely to report ever having had STIs compared to those residing <5 km from a healthcare clinic; those who are not receiving child support grants, those who are not smoking; and young women residing in Umgudulu. Similarly, young women with healthcare proximity between 5 and 9 km [aOR = 26.92; 95% (CI = 1.41–513.05)] who receive child support grants [aOR = 2.94; 95% (CI = 1.34–6.42)], drink alcohol [aOR = 1.07; 95% (CI = 0.49–2.34)], are from polygamous family structures [aOR = 2.41; 95% (CI = 1.02–5.69)], reside in Banana City [aOR = 3.32; 95% (CI = 1.12–9.81)] and are affiliated with the Xhosa tribe [aOR = 2.92; 95% (CI = 1.34–6.32)] were more likely to report having HIV compared to those residing <5 km from a healthcare clinic; young women who never receive child support grants; those who never drank alcohol; young women from monogamous family structures; young women residing in Umgudulu; and those who are affiliated with the isiZulu tribe. In the same vein, young women who had ever experienced GBV [aOR = 2.58; 95% (CI = 1.05–6.33)], who receive child support grants [aOR = 24.35; 95% (CI = 12.22–48.67)], those from polygamous family structures [aOR = 3.55; 95% (CI = 1.34–9.42)], young women residing in Banana City [aOR = 5.91; 95% (CI = 2.05–17.04)], and those affiliated with the Xhosa tribe [aOR = 1.97; 95% (CI = 0.98–3.98)] were more likely to have ever had an unintended pregnancy, compared to those who never experienced GBV, who never receive child support grants, those from monogamous family structures, those residing in Umgudulu and those affiliated with the isiZulu tribe.

On the other hand, young women dwelling in Durban informal settlements who discuss sexual matters with their parents were less likely to report having STIs [aOR = 0.18; 95% (CI = 0.10–2.74)] [aOR = 0.40; 95% (CI = 0.17–0.97)] or ever having had an unintended pregnancy [aOR = 0.03; 95% (CI = 0.01–2.11)] compared to those who never discussed sexual matters with their parents. In the same vein, young women living with their parents were less likely to report having STIs [aOR = 0.22; 95% (CI = 0.09–0.57)] or ever having had unintended pregnancy [aOR = 0.26; 95% (CI = 0.11–0.59)] compared to those not living with parents.

### Key Informant Interviews

The quantitative analysis results showed inequalities associated with adverse SRH outcomes among young women in Durban informal settlements. However, the researchers conducted key informant interviews to determine the desired interventions or solutions that the same population feels are required to bridge these inequalities. The authors thematically categorized the participants' responses in line with the quantitative study results as interventions required to reduce or eliminate the influence of (i) contextual factors, (ii) social inequality, (iii) patterns of behavior, and (iv)parental connectedness on adverse SRH outcomes among young women dwelling in Durban informal settlements.

### Characteristics of Participants

Key informant interviews were conducted with 20 key young women dwelling in the same informal settlements where the quantitative study was conducted. Five (5) participants were selected from each settlement.

The participants' mean age was 21.80 years, with a standard deviation of 2.17 years and age ranging from 18 to 24 years. All the participants were young women and were literate.

### Contextual Factors and Adverse Sexual and Reproductive Health Outcomes

Contextual factors were measured by gender-based violence and healthcare proximity. Both measures were associated with either of the adverse SRH outcomes.

#### Gender-Based Violence

- Yes, I have been raped. I was 11 years, and I was raped (Umgudulu, Participant 2).
- No, I have never been raped. However, when some guys come for me and ask for sex, I will give them willingly because I don't want them to force me to do it so I wouldn't call that rape (Umgudulu, Participant 4).

#### Suggested Solutions

- To stop the rape of young girls is for young girls to speak when they feel uncomfortable with their uncles, brothers and fathers, and some of the young girls should not wear short clothes around. Some young girls wear short clothes when their parents are not around, and they will be gallivanting around in short clothes. This could invite rape (Umgudulu, Participant 2).
- Young girls must speak up and allow them so that they wouldn't injure you or kill you, then report later (Umgudulu, Participant 4).

#### Healthcare Proximity

- There is no clinic around Banana City informal settlement. Family planning and other sexual and reproductive health services are gotten at Reservoir Hill clinic, which is around 10 min drive (Banana City, Participant 1).
- There is no clinic for family planning service here, we get it at Reddit Avenue at Reservoir Hill, and it's very far from here. Whenever I want to go, I get discouraged because of the distance (Umgudulu, Participant 2).

#### Suggested Solutions

- Having health clinic centers closer to the informal settlement, like mobile health facilities where required services and information on sexual education will be shared. Health experts should tell us what we need to do and what we are not doing right about contraception sexual and reproductive health, and the services should be free (Banana City, Participant 1).
- More mobile clinics should be established in places like informal settlements where Nurses should be routinely sent here, which will help us a lot (Umgudulu, Participant 2).

### Social Inequality and Adverse Sexual and Reproductive Health Outcomes

Social inequality was measured by the child support grant and wealth index. Only the child support grant was associated with SRH outcomes.

#### Social Inequalities Identified and Suggested Solutions

- Well, for me, the social grant shouldn't be given to children because there should be an age where a grant should be given to a baby. After all, you see a girl <16 years with a baby and then get the social grant, she will get another pregnant and get another money, but she will give these children to her parent and be using the money anyhow. I think it should be stopped (New Germany, Participant 1).
- Aahh! Yes, the social grant makes young girls to make more children, and if they increased it, it would make girls to have more children as well because they are having sex without a condom. I feel the government should stop it (Quarry Road, Participant 1).
- Some of them get pregnant because they want more money, and it's not right because R460 is not enough to raise a child, especially when the father impregnates the girl and runs away. Some of the young girls want to give birth to more children so that they can have a lot of money from the social grant, and it is not helping (Banana City, Participant 1).

### The Pattern of Behavior and Adverse Sexual and Reproductive Health Outcomes

The pattern of behavior was measured by alcohol intake and cigarette smoking.

#### Pattern of Behavior

- Let me make an instance for me. If I have taken alcohol, I won't use my brain; when a man makes a move, I won't think about it, and we will have sex without a condom or protection. It's the same thing when I am high on smokes or narcotics (Banana City, Participant 4).
- I don't think people who smoke or drink alcohol will have time to think of contraception or condom. A lot of times, it usually turns to one-night stands. I don't think they will think about condoms. Who cares about condoms in one-night stands? (New Germany, Participant 1).
- I don't think those who were drunk on high on smoke will use a condom because when I was drunk, I didn't use condoms because when a person is drunk, her mind is not working properly (Quarry Road, Participant 2).

#### Suggested Solution

- The government should work on limiting the use of alcohol and smoke among young women. If these can be reduced, it will reduce the non-use of condoms and other sexual and reproductive health services (Banana City, Participant 1).

### Parental Connectedness and Adverse Sexual and Reproductive Health Outcomes

#### Parental Connectedness

- Yes, if my parent had talked to me about puberty, sex, HIV/STIs, I would have used condoms, maybe I would do better, and maybe I wouldn't even have this child, but they don't talk to me about it (Quarry Road, Participant 5).
- If my parent had not told me about sexual health or puberty, I might still have used a condom because I learned these things from school, but I know that the information from school is not enough. However, if my parents talked to me about this, I would use a condom more consistently (Quarry Road, Participant 3).
- Because the girls don't have much information from their parents, they wouldn't want to use a condom (New Germany, Participant 5).
- Living with your parent helps make decisions about good sexual and reproductive health, such as using condoms, contraception, and unintended pregnancy. Many children here don't stay with their parents, and some of their parents drink a lot and do not attend to the young girl's needs or questions about sexual health (Banana City, Participant 1).

#### Suggested Solution

- I think when parents talk to their girls more, it will help them in their decision to use condoms or family planning. So, the parent should talk to their girls more and ensure they live together (New Germany, participant 4).

## Discussion

Accelerating the attainment of SDG target 3.7 requires evidence-based interventions. As such, the present study explored adverse SRH outcomes inequality among young women in Durban informal settlements, South Africa, while suggesting the required social and behavioral interventions to reduce the disparity. The quantitative analysis indicates that healthcare proximity, child support grants, cigarette smoking, alcohol consumption, polygamous family structures, GBV and residing in Banana City or belonging to the Xhosa tribe were associated with higher odds of reporting STIs HIV and unplanned pregnancies. However, these factors were associated with adverse SRH outcomes in varied ways.

Access to child support grants emerged as a significantly associated factor in relation to adverse SRH outcomes. The child support grant in South Africa was introduced in the late 1990s as an initiative to improve the quality of life of young women who become mothers through monthly state-funded cash transfers (45). In order for the young woman to be eligible for this grant, the child must be below age 18 and living with the biological parent(s) or primary caregiver. The study results indicate that young women who had access to child support grants reported higher odds of STIs, HIV, and unplanned pregnancies than those who did not have access. This result aligns with that of Jordan et al. (46), who reported that South African young women are getting pregnant in order to access child support grants. A plausible explanation for this observation could be that young women perceive the child support grant as an opportunity to make money. As such, instead of this intervention to reduce their sexual behaviors, it rather encourages them to engage in unprotected sex, thereby raising their risk of unplanned pregnancies, STIs, and HIV.

The findings also show that residing in Banana City was associated with higher odds of STIs, HIV and unplanned pregnancies. This result reflects the high level of poverty and deprivation in Banana City, as compared to Umgudulu. For instance, Phoku (47) reported that food insecurity and poverty are high in Banana City. Hence, young women will place less priority on having protected sex, thereby exacerbating their risk of STIs, HIV and unplanned pregnancies. Findings from the qualitative analysis support this association by linking the higher level of adverse SRH outcomes to the absence of clinic and family planning services in Banana City. Relatedly, the results suggest that young women who identified with the Xhosa tribe were more likely to have HIV or unplanned pregnancies than those who identified with isiZulu. This association could be linked to the Xhosa culture about contraception norms. In the Xhosa culture, public conversations about conception and family planning are taboo (48). As such, young women are denied accurate information about contraception, condom use and family planning. This inadvertently increases the risk of unprotected sex, which can exacerbate the risk of HIV and unplanned pregnancies.

Results from the quantitative analysis suggest that young women who smoked cigarettes and consumed alcohol were more likely to experience STIs and HIV, respectively, compared to those who did neither. This is in line with findings from the qualitative analysis where respondents revealed that people who smoke or drink alcohol do not have time to think of contraception or condoms. Hence, these women are more likely to engage in risky sexual behaviors, including concurrent multiple sexual partnerships, transactional sex and unprotected sex, elevating their odds of contracting STIs and HIV (48). Our findings also highlight the need to improve healthcare proximity in Durban informal settlements, as longer proximity to healthcare was associated with a greater risk of STIs and HIV.

The researchers also observed that young women in the polygamous family structure were more likely to experience unplanned pregnancy or HIV. The findings can be explained from the perspective that, in a polygamous family structure, there is heightened competition for scarce resources and insufficient parental control (49). Given that the family is the primary agency for the socialization of its members, these deficiencies associated with the polygamous family structure is likely to reduce women's capacity to make informed decisions such as practicing protected sex, negotiating for safe sex, etc., thereby contributing to an elevated risk of unplanned pregnancies and HIV among young women in this family structure. Women who had experienced GBV were at higher risk of unplanned pregnancy, which aligns with related studies conducted in South Africa (50–52). GBV that manifests through sexual abuse prevents young women from practicing or negotiating for safe sex, thereby putting them at higher risk of unplanned pregnancy.

Concerning parental connectedness and adverse SRH outcomes, our findings revealed that living with parents and communicating with parents about SRH issues was a significant protective factor that limited the risk of STIs and unplanned pregnancy. This result corroborates a related study that showed that the quality of parental connectedness significantly influenced condom and contraceptive use, which is necessary for reducing the risk of STIs and unplanned pregnancy among young women in informal settlements (38).

### Policy Implications

The findings from our study underscore the need for targeted interventions that focus primarily on young women who are at higher risk of adverse SRH outcomes. More specifically, the results highlight the urgency for the government to prioritize the SRH needs of informal settlements in Durban, particularly in Banana City. More social and health infrastructure (i.e., family planning service centers, clinics, etc.) would have to be developed in Durban's informal settlements, especially in Banana City. Public health educational promotions should be expanded to informal settlements in order to break socio-cultural norms that fuel GBV, which has proven to be significantly associated with adverse SRH outcomes. Our findings also iterate the need for the government to develop down strong policies and ensure the enforcement of already existing policies that control cigarette smoking and alcohol consumption among young people in South Africa.

### Research Implication

Our study has some implications for future research. In the future, researcher should consider a longitudinal design in order to be able to establish causal inferences between the various factors considered in this analysis and their relationship with adverse SRH outcomes overtime, researcher should also consider including young men in the future in order to give better view of how young men contributes to young women adverse SRH outcomes.

### Strengths and Limitations

The use of mixed methods in this study helped complement the shortcomings of both qualitative and quantitative methods. The researchers triangulated the data from the qualitative and quantitative study appropriately, ensuring the validity and reliability of the findings. However, there are some noteworthy limitations. Since the results of this study were self-reportedby the young women during the interview, there is possiblity of recall due to biases from the information supplied.

## Conclusion

Based on our findings, it is clear that there are disparities in the factors associated with adverse SRH outcomes in Durban's informal settlements. Healthcare proximity, child support grants, cigarette smoking, alcohol consumption, polygamous family structures, GBV, and residing in Banana City or belonging to the Xhosa tribe was associated with higher odds of reporting STIs, HIV and unplanned pregnancies. Living with a parent and communicating with parents about SRH issues were associated with a lower risk of adverse SRH outcomes.

## Coronavirus Rule and Guideline Compliances

Since data collection took place during the coronavirus disease (COVID-19) pandemic, the principal investigator ensured that all COVID-19 rules and guidelines were complied with as recommended by South Africa and the World Health Organization. Coronavirus adherence prescribed recommendations such as social distancing, wearing of face masks, frequent washing of both hands, and using alcohol-based sanitizer was highly upheld during the data collection and while having physical engagement or contact with any person.

## Data Availability Statement

The raw data supporting the conclusions of this article will be made available by the authors, without undue reservation.

## Ethics Statement

The University of KwaZulu-Natal's Ethical Review Body approved this study with Reference number: HSSREC/00002192/2020 before the questionnaire administration. The principal investigators and research assistants endeavored to provide participants with information on the purpose of the study, the process and how the findings will be used. The principle of voluntary participation guided the study, and, as such, each participant was required to sign an informed consent form. The participants were also promised that all ethical research considerations such as anonymity and confidentiality of the information provided would be upheld. All the institutional review board's guidelines for research using human subjects were also considered in this study.

## Author Contributions

OB developed the study's concept, conducted the primary data collection and key informant interviews, drafted the abstract, introduction, methodology, discussion, and conclusion sections, and analyzed the study. TB supervised the study at every stage and contributed substantially to the overall development of this study. All authors proofread the first draft of the manuscript and approved the final version for submission.

## Conflict of Interest

The authors declare that the research was conducted in the absence of any commercial or financial relationships that could be construed as a potential conflict of interest.

## Publisher's Note

All claims expressed in this article are solely those of the authors and do not necessarily represent those of their affiliated organizations, or those of the publisher, the editors and the reviewers. Any product that may be evaluated in this article, or claim that may be made by its manufacturer, is not guaranteed or endorsed by the publisher.

## Abbreviations

AGYW: Adolescent girls and young women
aOR: Adjusted odds ratio
cOR: crude odds ratio
GBV: Gender-based violence
HIV: Human immunodeficiency virus
ICPD-PoA: International Conference on Population and Development's Programme of Action
SDG: Sustainable development goal
SRH: Sexual and Reproductive Health
STIs: Sexually transmitted infections
SSA: Sub-Saharan Africa
MDGs: Millennium Development Goals
SADHS: South Africa Demographic and Health Survey
UN: United Nations.
