# Corrigendum: A mixed-method analysis of inequalities associated with adverse sexual and reproductive health outcomes and the requisite interventions among young women in Durban informal settlements, South Africa

**DOI:** 10.3389/fpubh.2023.1156307

**Published:** 2023-03-09

**Authors:** Obasanjo Afolabi Bolarinwa, Tlou Boikhutso

**Affiliations:** Department of Public Health Medicine, School of Nursing and Public Health, University of KwaZulu-Natal, Durban, South Africa

**Keywords:** mixed-method analysis, inequality, adverse sexual and reproductive health outcomes, informal settlements, South Africa

In the original article, there was a typo error in the figures reported for STI, HIV and unintended pregnancy.

A correction has been made to the **Abstract**, “*Results*” subsection and to the **Results** section, on page 1 paragraph 3 and page 4 paragraph 25 respectively, in which the incorrect figures were erroneously reported. These sentences should be corrected as follows:

**Abstract**

“At the quantitative level, the prevalence of adverse SRH outcomes among young women dwelling in Durban informal 242 settlements were 17.55%, 9.14% and 18.10% for STIs, HIV and unintended pregnancy, respectively.”

**Results**:

“The prevalence of adverse SRH outcomes among young women dwelling in Durban informal 242 settlements were 17.55%, 9.14% and 18.10% for STIs, HIV and unintended pregnancy, respectively.”

The authors apologize for these errors and state that this does not change the scientific conclusions of the article in any way. The original article has been updated.

